# Aberrant MicroRNAs in Pancreatic Cancer: Researches and Clinical Implications

**DOI:** 10.1155/2014/386561

**Published:** 2014-05-08

**Authors:** Tao Sun, Xiangyu Kong, Yiqi Du, Zhaoshen Li

**Affiliations:** Department of Gastroenterology, Changhai Hospital, Second Military Medical University, 168 Changhai Road, Shanghai 200433, China

## Abstract

Pancreatic ductal adenocarcinoma (PDAC) is an aggressive malignancy with a high rate of mortality and poor prognosis. Numerous studies have proved that microRNA (miRNA) may play a vital role in a wide range of malignancies, including PDAC, and dysregulated miRNAs, including circulating miRNAs, are associated with PDAC proliferation, invasion, chemosensitivity, and radiosensitivity, as well as prognosis. Greater understanding of the roles of miRNAs in PDAC could provide insights into this disease and identify potential diagnostic markers and therapeutic targets. The current review focuses on recent advances with respect to the roles of miRNAs in PDAC and their practical value.

## 1. Introduction

Pancreatic ductal adenocarcinoma (PDAC) is highly malignant and has a poor prognosis. The overall 5-year survival rate for PDAC is less than 5% [1]. Researchers estimated that about 45,220 cases of PDAC were newly diagnosed and 38,460 PC-related deaths occurred in the United States in 2013 [2]. Surgery remains the best choice for PDAC treatment. However, most patients are diagnosed at an advanced stage, making them poor candidates for surgical resection. Lack of early alarming symptoms, rapid local or distant metastasis, highly malignant phenotypes, and innate resistance to conventional chemotherapeutics are the major reasons for the dismal prognosis for PDAC. Therefore, there is an urgent need to develop new diagnostic strategies and prognostic markers as well as potential therapeutic targets to improve the outcome of PDAC patients.

Since first discovered in* Caenorhabditis elegans* in 1993, microRNAs (miRNAs) have unraveled new mechanisms for regulation of gene expression and have provided new directions for cancer research. miRNAs are comprised of a class of highly conserved short noncoding, 17–25 nucleotide long RNA products [3] that regulate gene expression at the posttranscriptional level. They are negative regulators of gene expression through base pair interactions with the 3′ untranslated region (3′UTR) of protein-coding mRNAs. Partial complementarity between the miRNAs and the 3′UTR of the target transcripts leads to inhibition of translation, while perfect complementarity results in degradation of mRNAs [4]. miRNAs are predicted to regulate the activity or gene expression of over 30% of all protein-coding genes in mammals. So far, more than 1800 human miRNAs have been identified [5–8].

Since the discovery of miRNA's involvement in chronic lymphocytic leukemia [9], tremendous studies have validated the fact that aberrant expression of miRNAs is associated with cancers [10–14]. Extensive mapping of miRNA genes showed that these oncomiRs are often located at genomic regions associated with cancer. Previous studies have reported that while elevated expression of some miRNAs is associated with carcinogenesis (oncogenes), others may inhibit cancer by reducing cell proliferation, survival, and cellular differentiation [15]. miRNA expression profiling signatures can distinguish cancer from benign tissues and this may provide the basis for developing new diagnostic and therapeutic strategies [16].

The number of studies on miRNAs in a PDAC setting is increasing at an exponential rate in recent years. However, the potential clinical use of miRNAs in diagnosis and treatment of PDAC and their prognostic value have not been well summarized yet. The present review focuses on recent advances of miRNA research in PDAC and their potential practical value.

## 2. Aberrant miRNA Expression Patterns in PDAC

In recent years, numerous approaches have been developed to quantify miRNA levels [17–19]. These approaches have identified distinct cell- and tissue-specific miRNA expression patterns in PDAC specimens as compared with controls (Table 1). The earliest report regarding pancreas showed that miR-375 and miR-376 were expressed at higher levels in mouse pancreas and pancreatic islet cells than in mouse brain, heart, and liver tissues [20]. Following studies showed that the expression of miR-376 precursor in PDAC cell line PANC-1 was among the highest of all cell lines studied, while expression of miR-375 in the two PDAC cell lines studied did not differ from the other cell lines [21].

Accumulating efforts were then made to explore the miRNA expression signature that is associated with PDAC. Employing RT-PCR, Eun et al. profiled more than 200 miRNA precursors in specimens of human PDAC, paired benign tissue, and normal pancreas. One hundred miRNA precursors were aberrantly expressed in PDAC or desmoplasia, including miRNAs that were previously reported in other human cancers, for example, miR-21, miR-155, miR-221, miR-222, and miR-424-5p, as well as those that were not previously reported in cancers, for example, miR-376a and miR-301. Most of the top aberrantly expressed miRNAs displayed increased expression in tumors. Reverse transcription in situ PCR showed that three of the top differentially expressed miRNAs (miR-221, miR-376a, and miR-301) were localized in tumor cells but not in stroma, normal acini, or ducts [28]. In another study, Bloomston et al. compared the global miRNA expression pattern of resected pancreatic cancer with matched benign adjacent pancreatic tissue and chronic pancreatitis. Specimens were obtained from microdissected paraffin blocks. The miRNA microarray result demonstrated that twenty-one miRNAs with increased expression and 4 with decreased expression were identified and correctly differentiated pancreatic cancer from benign pancreatic tissue in 90% of samples by cross-validation. Fifteen overexpressed and 8 underexpressed miRNAs differentiated pancreatic cancer from chronic pancreatitis with 93% accuracy. Upregulation of miR-155, miR-181a,b,c,d, miR-21, miR-196a, and miR-221 and downregulation of miR-148a,b and miR-375 could differentiate PDAC from normal pancreas and pancreatitis tissue samples [22]. Quantitative RT-PCR was used to confirm the findings of the microarray.

Some of the aberrant miRNAs reported by those studies may play an important role in genesis and metastasis of PDAC. Overexpression of miR-221 may be essential for the platelet-derived growth factor (PDGF)-mediated epithelial-mesenchymal transition phenotype, migration, and growth of pancreatic cancer cells [29]. The mRNA expression level of sel-1-like (SEL1L), a tumor suppressor gene, was found to correlate inversely with the expression of hsa-mir-143, hsa-mir-155, and hsa-mir-223 [31]. Functional analysis revealed that hsa-mir-155 acted as a suppressor of SEL1L in PDAC cell lines. Wu et al. confirmed the upregulation of miR-424-5p expression level in PDAC cells by quantitative RT-PCR and found that the high expression of miR-424-5p suppressed the expression of cytokine-induced signaling 6 (SOCS6), leading to increased proliferation, migration and invasion of pancreatic cancer cells, and inhibited cell apoptosis [37]. Zhang et al. identified cholecystokinin-B receptor (CCKBR) and B cell lymphoma (Bcl-2) as targets of miR-148a, which acted as a tumor suppressor, in the regulation of pancreatic cancer growth and apoptosis [43].

Szafranska et al. investigated the expression of 377 miRNAs in snap-frozen surgical resection pancreatic tissue samples from normal pancreas, chronic pancreatitis (CP), and PDAC. A pancreatic miRNome was established by miRNA arrays and confirmed by quantitative RT-PCR. They found that the expression of some miRNAs, such as miR-29c, miR-96, miR-143, miR-148b, and miR-150, was dysregulated in both CP and PDAC samples, whereas miR-196a, miR-196b, miR-203, miR-210, miR-222, miR-216, miR-217, and miR-375 were aberrantly expressed only in the PDAC samples. The authors concluded that a combination of miR-217 and -196a was able to discriminate normal pancreas, CP, and cancerous tissues [33]. Zhang et al. analyzed miRNA expression of 10 pancreatic cancer cell lines and 17 pairs of pancreatic cancer/normal tissue. The author reported that eight miRNAs, including miR-196a, miR-221, and miR-222, were significantly upregulated in most PDAC tissues and cell lines. The incidence of upregulation of these eight genes between normal control subjects and tumor cells or tissues ranged from 70% to 100% [34].

Ohuchida et al. obtained the miRNA expression profiles of pancreatic cancer cell line CAPAN-1 by microarray analysis. Compared with immortalized human pancreatic ductal epithelial cell line, 8 miRNAs (including miR-10a, miR-17-5p, miR-92, et al.) were upregulated and 2 miRNAs (miR-450 and miR205) were downregulated in CAPAN-1 cells. The microarray data was then confirmed with quantitative RT-PCR analysis. Microdissection analyses revealed that miR-10a was overexpressed in pancreatic cancer cells isolated from a subset of primary tumors (12 of 20, 60%) compared with precursor lesions and normal ducts. And further vitro experiments demonstrated that miR-10a may be involved in the invasive potential of PDAC cells partially via suppression of HOXA1 [38].

In a recent study, Zhang et al. reported a novel mechanism through which increased zinc mediated by the zinc importer ZIP4 could transcriptionally upregulate the expression level of miR-373 in PDAC cells to promote tumour growth. Higher expression of miR-373 was regulated by the zinc-dependent transcription factor CREB, and it enhanced cell proliferation, invasion, and tumour growth through negative regulations against TP53INP1, LATS2, and CD44 [39].

Pancreatic cancer is characterized by a dense stromal reaction. There is accumulating evidence that pancreatic stellate cells (PSCs) promote the progression of pancreatic cancer. In a study focusing on the relationship between PSCs and PDAC, the expression level of miR-210 in PDAC cells was significantly induced by coculturing with PSCs [41]. This upregulation may be attenuated by inhibition of ERK and PI3K/Akt pathways, and the inhibition of miR-210 expression decreased migration, decreased the expression of vimentin and snail-1, and increased the membrane-associated expression of *β*-catenin in PANC-1 cells through coculturing with PSCs.

Panarelli's studies evaluated miRNA expression in pancreatic resection specimens and fine- needle aspiration biopsies. PDAC showed a higher expression of miR-21, miR-221, miR-155, miR-100, and miR-181b than benign lesions (intraductal papillary mucinous neoplasms and nonneoplastic tissues) by qRT-PCR. Microarray analysis of a subset of carcinomas and intraductal papillary mucinous neoplasms confirmed overexpression of miR-21, miR-221, and miR-181b. Cell blocks containing carcinoma showed higher expression of miR-21, miR-221, and miR-196a than those from benign lesions. These results indicated that a select panel of miRNAs may aid in the distinction among pancreatic lesions in cytology specimens [23].

The majority of PDAC overexpress mesothelin (MSLN), which contributes to enhanced proliferation, invasion, and migration. Marin-Muller et al. compared the expression of 95 cancer-associated miRNAs of PDAC cells with overexpressed or low endogenous MSLN levels. RT-PCR result showed a global dysregulation of miRNA expression, with several miRNAs either upregulated (i.e., miR-10b and miR-196a) or downregulated (i.e., miR-198, miR-200c, and miR-155). miR-198 was the most downregulated in PDAC by overexpression of mesothelin, through NF-*κ*B-mediated OCT-2 induction [45]. The authors suggested that miR-198 acts as a central tumor suppressor and modulates the molecular makeup of a critical interactome in PDAC. Reconstitution of miR-198 in pancreatic cancer cells results in reduced tumor growth, metastasis, and increased survival through directly targeting MSLN, PBX-1, and VCP.

Recent explosion in the knowledge of the molecular genesis regarding pancreatic cancer has defined PDAC as a disease with alterations of a wide range of signaling cascades, which is in contrast with certain tumors that are driven by a single oncogene. However, certain signaling pathways and key nodal points act as core genetic alterations and are commonly detected in PDAC cells. Since miRNA alteration is quite informative in pancreatic cancer diagnosis, exploring those commonly deregulated miRNAs and their targeting proteins will help identify those potential targets for future therapy. The miRNAs most frequently reported in the literature exhibiting aberrant expression in PDAC were miR-15b, miR-21, miR-146a, miR-155, miR-181b, miR-196a, miR-200, and miR-221/222 [21, 22, 28, 33, 34, 46] (Table 1).

## 3. Biomarkers for Early Detection of PDAC

It is generally recognized that PDAC is an insidious disease with no specific early clinical symptoms, except when the primary tumor is located in the head of the pancreas (obstructive jaundice). A longer interval between the onset of symptoms and the initial diagnosis of PDAC is associated with the disease being first identified at a more advanced stage with poor prognosis. At the time of diagnosis, less than 15% of patients come with surgically resectable disease. The median survival of unresectable PDAC is only 4–6 months. Although the overall 5-year survival of large resected PDAC (median size 3 cm) is only 10%–20%, it is 30%–60% after resection of small PDAC (tumor size ≤ 2 cm) and exceeds 75% when minute PDAC (≤10 mm in size) is resected [52].

Early detection of PDAC would logically require the detection of small lesions. Current noninvasive imaging techniques such as ultrasound, contrast-enhanced multidetector computed tomography, and magnetic resonance imaging are inadequate for the detection of PDAC at an early stage, because they could not reliably detect tumors <1-2 cm in size [53]. Even invasive techniques, such as endoscopic retrograde cholangiopancreatography (ERCP) and endoscopic-ultrasound (EUS) guided fine-needle aspiration (FNA), which are used to distinguish foci of malignant change in the background of CP, present difficulties.

Serum and plasma remain the most easily accessible samples for diagnostic testing and, hence, are an attractive medium for biomarker testing to screen early-stage diseases. To date, the clinical role of PDAC markers for diagnosis is still limited; meanwhile, the development of minimally invasive biomarker assays for early detection is urgently needed. Several reports indicated that miRNA expression profiles may be useful in diagnosis of specific cancer types [15, 54–63]. For example, Lawrie et al. found that circulating miR-21 was significantly overexpressed in sera from diffuse large B cell lymphoma patients. High expression levels of miR-21 were found to be associated with improved relapse-free survival times.

Kong et al. found that three serum miRNAs, including miR-196a, were differentially expressed in PDAC compared with control groups. Serum miR-196a could be a potential noninvasive marker for PDAC prognosis and selection for laparotomy [35]. Another investigation by Wang et al. showed that the expression levels of four miRNAs in plasma—miR-21, miR-210, miR-155, and miR-196a—were significantly higher in patients with PDAC than in a healthy control group [24].

Li et al. measured 735 circulating miRNAs in PDAC case and control sera [64]. miR-1290 was found to show the best diagnostic performance among all the significantly elevated circulating miRNAs, yielding an area under curve (AUC) of 0.96 [95% confidence interval (CI), 0.91–1.00], 0.81 (0.71–0.91), and 0.80 (0.67–0.93), for subjects with pancreatic cancer relative to healthy controls, subjects with chronic pancreatitis, and pancreatic neuroendocrine tumors, respectively. Kawaguchi et al. found that plasma miR-221 concentrations were significantly higher in PDAC patients than those in benign pancreatic tumors and controls [30]. Furthermore, PDAC patients with high plasma miR-221 concentrations showed significant correlation with distant metastasis and nonresectable status.

Early diagnosis for PDAC requires markers with high sensitivity and specificity. The standard serum marker, salivated Lewis blood group antigen CA19-9, is widely used, but its use is limited to monitoring responses to therapy, not as a diagnostic marker [65, 66]. Recent research results from our group indicate that sera or plasma from patients with PDAC has a unique miRNA expression pattern compared with normal control as well as CP [67, 68]. The combination of miR-16, miR-196a, and CA19-9 was more effective for discriminating PDAC from non-PDAC (normal and CP) with a sensitivity of 92.0% and a specificity of 95.6%. These studies suggest that the amount of miRNAs in serum may have the potential as diagnostic biomarkers for PDAC [35]. miR-210 also has been detected in sera of PDAC patients, while it was expressed at levels that were fourfold higher than in normal controls [42].

Wang et al. [69] investigated miRNA expression in peripheral blood mononuclear cells (PBMCs) in healthy, benign pancreatic/peripancreatic disease (BPD) and PDAC cohort, respectively. Using the method of sequencing technology and quantitative RT-PCR, they found that miR-27a-3p level in PBMCs could discriminate PDAC from BPD. Frampton et al. further examined the miRNA profiles in PBMCs from PDAC patients, based on the theory that circulating blood cells monitors the patients' physiological state and response by altering their transcriptome [70]. They confirmed that miR-27a-3p was upregulated in PBMCs isolated from PDAC patient blood samples and the combination of PBMC miR-27a-3p with serum CA19-9 levels improved diagnostic accuracy.

Sometimes it can be difficult to distinguish malignant and benign lesion on the pancreas with conventional imaging techniques such as CT (computed tomography), MRI (magnetic resonance imaging), and abdominal B-ultrasound. The accuracy of endoscopic ultrasound-guided fine-needle aspiration (EUS-FNA) biopsy is always affected by the location and size of the lesion, the quantity of tissue obtained, the quality of the histology, et al. A molecular analysis of miRNA expression may improve the diagnosis accuracy. Szafranska et al. used qRT-PCR to quantify miRNA levels in FNA samples and compared the results with a training set consisting of frozen macrodissected pancreatic samples. The authors reported that a combination of miR-196a and miR-217 biomarkers had the ability to distinguish between healthy tissue, PDAC, and CP in the training set as well as segregate PDAC FNA samples from other FNA samples [36]. Hanoun et al. measured the level of DNA methylation of EUS-FNA samples from PDAC and CP patients. Hypermethylation of the DNA region encoding miR-148a led to the inhibition of its gene expression in preneoplastic pancreatic intraepithelial neoplasia (PanIN) [44]. The authors suggested that this phenomenon of hypermethylation can differentiate PDAC from CP and the hypermethylated DNA region encoding miR-148a can serve as an ancillary marker for the differential diagnosis of PDAC and CP.

In some recent studies, miRNAs are also found to be useful as diagnostic markers for some precursor lesions of PDAC. Caponi et al. quantified the expression of three candidate miRNAs (miR-21, miR-155, and miR-101) by quantitative RT-PCR in 86 laser-microdissected intraductal papillary mucinous neoplasms (IPMNs) specimens [71]. They found that miR-21 and miR-155 were upregulated in invasive IPMNs compared with noninvasive IPMNs and in noninvasive IPMNs compared with normal tissues. However, miR-101 levels were significantly higher in noninvasive IPMNs and normal tissues compared with invasive IPMNs. Further multivariate analysis showed that high-miR-21 expression emerged as an independent prognostic biomarker in invasive IPMNs with bad survival. Lubezky et al. [72] also found miRNAs useful to identify IPMN with high risk for malignant transformation. They analyzed the expression patterns of 846 human miRNAs with microRNA microarray in 55 tissues that range from low-grade dysplastic IPMN to PDAC. Expression of 15 miRNAs, including miR-217, miR-216a, miR-21, and miR-155, was significantly different between two IPMN subgroups: low- and moderate-grade dysplastic IPMNs versus high-grade dysplastic IPMN and invasive cancer with IPMN. Pancreatic cysts are a group of lesions with heterogeneous malignant potential. Farrell et al. [73] compared the expression of miRNAs in benign, premalignant, and malignant cysts fluidusing a whole-genome expression array analysis. The results showed that pancreatic cyst fluids miR-21 and miR-221 are associated with invasive cancer. miR-221 was expressed at significantly higher levels in malignant cysts compared with benign or premalignant cysts and miR-21 was also expressed at significantly higher levels in premalignant and malignant cysts.

Despite the fact that the diagnostic value of miRNAs expression aberration in PDAC has been extensively studied in recent years, differences in measurement platforms and lab protocols can render gene expression levels incomparable. Ma et al. [74] in their recent metareview, which included a total of 538 tumors and 206 noncancerous control samples, identified a statistically significant miRNA metasignature of seven up- (including miR-21, miR-155, and miR-221) and three downregulated miRNAs (miR-217, miR-148a, and miR-375).

In conclusion, no PDAC marker has been shown to be useful in the early detection of an asymptomatic population so far. Serum and plasma miRNAs, for example, miR-21, miR-155, miR-210, and miR-196a, are promising biomarkers for early detection of PDAC, especially with the combination of serum CA19-9 levels.

## 4. Prognosis and miRNAs

Poor survival is a hallmark feature of PDAC. Several studies have suggested prognostic significance of miRNAs expression profiles in PDAC. For example, miR-21 appears to confer chemoresistance to PDAC cell lines; strong miR-21 expression was predictive of poorer outcomes compared with absent or faint/focal miR-21 expression in patients with node-negative PDAC (median 15.2 versus 27.7 months) [25]. Jamieson et al. [75] also found that miR-21 was associated with poor prognosis. In their study, they performed the global miRNA microarray expression profiling of frozen PDAC tissue from 48 patients confirmed by RT-PCR analysis. After a further validation set of 24 patients, they found that high expression of miR-21 and reduced expression of miR-34a were significantly associated with poor overall survival. Frampton et al. [76] in their recent study found that, in 91 PDAC samples from patients, high level of a combination of miR-21, miR-23a, and miR-27a was associated with shorter survival times after surgical resection.

miR-200c, a member of the miR-200 family, may be a valuable prognostic marker for PDAC. The expression of miR-200c in PDAC shows a wide range. While strong expression of miR-21 predicts limited survival in PDAC patients, high expression of miR-200c is a sign of good prognosis [49]. Specifically, the median survival times and five-year survival rates were 42 months and 33.5% in the high miR-200c expression group and 19 months and 11.2% in the low miR-200c expression group. In a recent study, researchers suggested that miR-200c overexpression downregulates transmembrane mucins MUC4 and MUC16 in pancreatic cancer cells by directly targeting the mRNA coding sequence of each, resulting in reduced levels of MUC4 and MUC16 mRNA and protein, which are associated with tumor progression and metastatic potential in human PDAC [50].

Elevated levels of miR-155, miR-203, miR-210, and miR-222 expression in PDCA were significantly associated with increased risk (6.2-fold) of death compared to patients with reduced expression of these miRNAs [32]. A subgroup of 6 miRNAs (miR-452, miR-105, miR-127, miR-518a-2, miR-187, and miR-30a-3p) was found to be able to distinguish long-term survivors with node-positive disease from those dying within 24 months [22]. miR-196a-2 may also be a negative survival predictor; median survival for pancreatic cancer patients has been shown to be 14.3 versus 26.5 months, depending on miR-196a-2 expression level. Elevated expression of miR-196a-2 was predictive of median survival differing by about a year among pancreatic cancer patients. In addition, increased expression of miR-155, miR-203, miR-210, and miR-222 was also found to be significantly associated with poorer survival of PDAC patients [32, 77]. Zhu et al. reported that reduced miR-218 in PDAC tissues was correlated with tumor progression and might be an independent poor prognostic factor for patients [78]. In a recent study, Zhao et al. found that miR-130b was significantly downregulated in 52 pancreatic cancer tissues (compared with paracancerous tissues) and five cell lines [79]. Furthermore, the deregulated miR-130b was correlated with worse prognosis, increased tumor size, late TNM stage, lymphatic invasion, and distant metastasis. Dual luciferase assay revealed that STAT3 may be one direct target of miR-130b.

## 5. Chemosensitivity and Radiosensitivity

**Figure 1:**
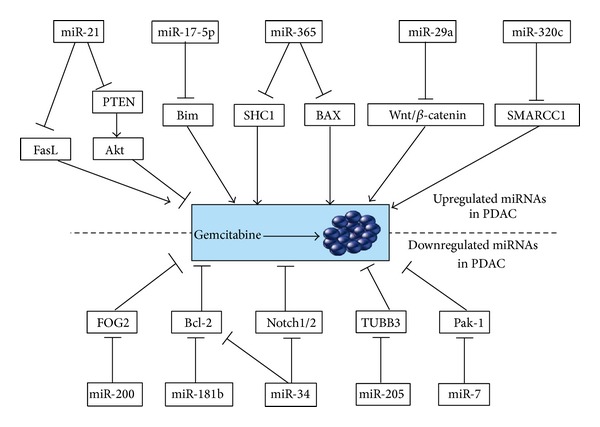
Influences of miRNAs on the gemcitabine treatment in PDAC. miRNAs that are upregulated in PDAC inhibit gemcitabine-sensitive associated genes, such as PTEN, Bim, SHC1, BAX, and SMARCC1. Conversely, miRNAs that are downregulated in tumors inhibit gemcitabine-resistant associated genes, such as Pak-1, TUBB3, Notch1/2, Bcl-2, and FOG2.

miRNAs have been shown to induce changes in the chemosensitivity or radiosensitivity of PDAC cells in a variety of settings, with certain miRNAs identified to indicators for chemotherapy efficacy (Figure 1). miR-21, for example, appears to convey chemoresistance to PDAC cells. miR-21 previously has been shown to be significantly upregulated in PDAC, whereas stronger expression of miR-21 is associated with poorer survival of this cancer [25], indicating its oncogenic properties. Forced expression of miR-21 increases proliferation and invasion of PDAC cell lines, and this appears to occur through target inhibition of the phosphatase and tensin homolog (PTEN), programmed cell death 4 (PDCD4), presence of tropomyosin 1 (TPM1), and tissue inhibition of metalloproteinases-3 (TIMP3), thereby indirectly inducing expression of matrix metalloproteinase-2 and -9 and vascular endothelial growth factor (VEGF). Meanwhile, miR-21 appears also to induce chemoresistance to gemcitabine in PDAC cell lines. For example, when PDAC cell lines PANC-1, LPc111, and LPc006 were transfected with the miR-21 precursor, those cells were resistant to gemcitabine treatment, showing reduced apoptosis and increased proliferation [26, 80]. In contrast, the inhibition of miR-21 function induced more apoptosis and decreased proliferation in PDAC cell line SUIT-2, which expresses relatively high levels of miR-21 [26]. In another study, Hwang et al. [27] suggested that miR-21 might be a useful biomarker for chemosensitivity, as their research showed that the PDAC cells with lower miR-21 expression had higher chemosensitivity to 5-fluorouracil (5-FU). miR-21 leads to downregulation of PTEN and a more active signaling through the PI3K/Akt/mTOR pathway. Modulation of apoptosis, Akt phosphorylation, and expression of genes involved in invasive behavior may contribute to the role of miR-21 in gemcitabine chemoresistance. Wang et al. confirmed that FasL was a direct target of miR-21. They found that increased FasL expression following gemcitabine treatment could induce cancer cell apoptosis, whereas the ectopic expression of miR-21 partially protected the cancer cells from gemcitabine-induced apoptosis [81].

Hamada et al. found that miR-365 was highly expressed in invasive PDAC and could induce gemcitabine resistance in pancreatic cancer cells. The authors suggested that miR-365 may induce chemoresistance through directly targeting adaptor protein Src homology 2 domain containing 1 (SHC1) and apoptosis-promoting protein BAX [82]. The siRNA-based knockdown of SHC1 and BAX increased gemcitabine resistance, indicating the miR-365/SHC1/BAX axis might influence the survival of pancreatic cancer cells.

Nagano et al. investigated the relationship between miR-29a expression and the response to gemcitabine in PDAC cells [83]. MIAPaCa-2 and PSN-1 cells transfected with anti-miR-29a showed significantly lower resistance to gemcitabine. Putative target molecules showed overexpression in the transfected cells including Dkk1, Kremen2, and sFRP2 and lower activation of the Wnt/beta-catenin signaling pathway. The authors suggested that activation of the Wnt/beta-catenin signaling pathway mediated the miR-29a-induced resistance to gemcitabine in PDAC cell lines

Iwagami et al. reported that high expression of miR-320c in MiaPaCa2 induced resistance to gemcitabine [84]. miR-320c-related resistance to gemcitabine was mediated through SMARCC1, a core subunit of the switch/sucrose nonfermentable (SWI/SNF) chromatin remodeling complex. Further clinical examination revealed that only SMARCC1-positive patients benefited from gemcitabine therapy with regard to survival after recurrence.

In a recent study, Yan et al. reported that transfected PDAC cell lines Panc-1 and BxPC3 with miR-17-5p inhibitor showed growth inhibition, spontaneous apoptosis, higher caspase-3 activation, and increased chemosensitivity to gemcitabine [85]. miR-17-5p inhibitor upregulated Bim protein expression in a dose-dependent manner without changing the Bim mRNA level, proving that miR-17-5p negatively regulates Bim at the posttranscriptional level.

miR-200 is a potential tumor suppressor that plays important roles in cancer metastases [86, 87]. Like miR-21, miR-200 may be involved in chemoresistance, or in this case chemosensitivity. Ali et al. reported that decreased expression of miR-200 and increased expression of miR-21 are associated with gemcitabine resistance in PDAC cells. Interestingly, treatment with curcumin, a major chemical component in turmeric, a spice commonly used in Indian cooking, resulted in upregulation of miR-200 expression and downregulation of miR-21 expression [88].

Several studies identified miRNAs which may sensitize PDAC to chemotherapy or radiotherapy. Although gemcitabine-resistant PDAC cell sublines (SW1990/GR and CFPAC-1/GR) expressed higher levels of miRNA-181b, Cai et al. found that gemcitabine induced higher levels of apoptosis in PDAC cells transfected with miRNA-181b mimics [89]. Nude mouse xenograft assay data showed that miR-181b transfection also sensitized the cells to gemcitabine treatment in vivo. Further study showed a reduced BCL-2 expression following miR-181b transfection but an enhanced caspase-3 activity in miRNA-181b mimic-transfected PDAC cells, indicating that miRNA-181b may sensitize PDAC cells to gemcitabine by targeting BCL-2. The study by Singh et al. identifies a series of miRNAs which were either upregulated (e.g., miR-146) or downregulated (e.g., miR-205, miR-7) in gemcitabine resistant MIA PaCa-2 cancer cells and clinical metastatic pancreatic cancer tissues [90]. Transfection with miR-205 resulted in the restoration of chemosensitivity to gemcitabine with decreased expression of stem cell markers OCT3/4 and CD44 and chemoresistance marker class III b-tubulin. miR-34 also appears to sensitize PDAC cells to chemotherapy and radiotherapy. It has been reported that expression of miR-34, which shows tumor suppressive qualities, is significantly lower in PDAC cell lines than in normal pancreatic ductal epithelial cell lines [91]. miR-34 expression normally is regulated by the tumor suppressor gene p53 [92], but it also can be inactivated by aberrant CpG methylation in cancer [93]. Evidences showed that restoration of miR-34 expression induces a G1 cell cycle arrest and apoptosis in some malignancies, including PDAC. In another study, Wang et al. found that miR-23b overexpression inhibited radiation-induced autophagy and sensitized PDAC cells to radiation. They suggested that, in PDAC, reduced miR-23b level increases levels of its target AGT12 and autophagy to promote radioresistance [94].

## 6. miRNAs as Potential Therapeutic Targets in PDAC

As discussed above, many miRNAs downregulate genes that are highly relevant to PDAC and contribute to disease progression; thus, chemically modified antisense oligonucleotides or ectopic expression of miRNAs might be considered for therapy. Since one single miRNA might potentially affect several target genes, artificially increasing or decreasing the expression signature of a given miRNA offers interesting therapeutic possibilities.

RNA interference (RNAi) was identified in* C. elegans* in 1998 [95] and in mammalian cells in 2001 [96]. Since then, RNAi has generated increasing interest and publications in diverse research areas. The main problem involved in RNAi-based gene therapy is the delivery of the effector molecule, which should preferably be controllable, sustained, and tissue-specific. Several groups have opted for nonviral delivery of synthetic miRNA molecules. miRNA mimics or miRNA antagomirs can be repeatedly delivered locally or systemically, causing transient suppression of target gene expression [97]. Morrissey et al. intravenously injected mice carrying replicating HBV with a stabilized siRNA targeting the HBV RNA that had been incorporated into a specialized liposome to form a stable nucleic-acid-lipid particle (SNALP). The improved efficacy of siRNA-SNALP was compared with unformulated siRNA leading to a longer half-life in plasma and liver. RNAi incorporated into SNALPs could protect them from degradation, prevent immunostimulation, and facilitate their uptake in endosomes [98]. In addition, 2′O-methyl modifications increase the stability of synthetic molecules, preventing off-targeting [99].

Aberrant miRNA expression in PDAC oncogenically affects cancer suppressor genes, causing subsequent effects on PDAC cell proliferation, apoptosis, and metastasis. For example, Tsuda et al. found that miRNA (Gli-1-miRNA-3548) and its corresponding duplex (Duplex-3548) significantly inhibited proliferation of Gli-1^+^ ovarian (SK-OV-3) and pancreatic (MiaPaCa-2) tumor cells. The miRNAs mediated delayed cell division and activation of late apoptosis in MiaPaCa-2 cells [100, 101]. miR-96 directly targets the KRAS oncogene, and ectopic expression of miR-96 can reduce pancreatic cell proliferation, migration, and invasion, suggesting its therapeutic potential in PDAC [48].

Other miRNAs with oncogenic or tumor suppressor functions, including let-7, miR-21, miR-27a, miR-31, miR-200, and miR-221, could be used as novel therapeutic agents for PDAC. Several studies reported that antisense to miR-21 and miR-221 could improve the chemosensitivity of gemcitabine, and the antisense-gemcitabine combinations resulted in significant cell killing under various conditions [26, 102]. Overexpression of miR-204, either by a miR-204 mimic or by triptolide treatment, downregulates myeloid cell leukemia-1 (Mcl-1) by directly binding to the Mcl-1 3′ UTR and causes a subsequent decrease in cell viability and pancreatic cancer cell death [103]. Yan et al. reported that miR-20a could regulate Stat3 at the posttranscriptional level, resulting in inhibition of cell proliferation and invasion of pancreatic carcinoma [47].

Both the inhibition of miR-31 in AsPC-1 and HPAF-II PDAC cells with high endogenous expression and forced expression of miR-31 in MIA PaCa-2 with low endogenous levels led to reduced cell proliferation, migration, and invasion. More importantly, in AsPC-1 cells, further enhancement of miR-31 also resulted in reduced cell migration and invasion, implicating that the level of miR-31 is critical for these phenotypes [104]. miR-27a may play an oncogenic role by targeting Spry2 and modulating the malignant behaviors of PDAC cells. Spry2 protein, which has a low expression level in pancreatic adenocarcinoma, was upregulated by transfection with a miR-27a inhibitor [40]. Torrisani et al. found that let-7 expression is strongly reduced in PDAC samples (compared with adjacent tissues). Restoring let-7 levels in cancer-derived cell lines, by transfection with plasmid-based synthetic miRNAs or by lentiviral transduction, strongly inhibited cell proliferation, K-ras expression, and mitogen-activated protein kinase activation. However, intratumoral gene transfer or implantation of Capan-1 cells stably overexpressing let-7 failed to impede tumor growth progression [51].

Frampton et al. analyzed the combined effects of altered activities of miRNAs in PDAC cell lines and in PDAC samples from patients. They found that 3 miRNAs (miR-21, miR-23a, and miR-27a) may act as cooperative repressors of a network of tumor suppressor genes that included PDCD4, BTG2, and NEDD4L. Inhibition of miR-21, miR-23A, and miR-27A had synergistic effects in reducing proliferation of PDAC cells in culture and growth of xenograft tumors in mice. The level of inhibition was greater than that of inhibition of miR-21 alone [76].

These studies opened a new perspective and provided early steps for miRNA replacement therapy for PDAC. However, before miRNA based therapeutics enter clinics, hurdle issues such as specific delivery to certain cells of interest, safety, and pharmacokinetics are warranted to be addressed.

## 7. Conclusion

It is well established that miRNAs are vital factors in a wide variety of biological processes, including development, cellular proliferation, invasion, and apoptosis. In PDAC, miRNAs showed aberrant processing and expression signatures. Identification of unique patterns of dysregulated miRNA expression in PDAC provides valuable information that may serve as molecular biomarkers for tumor diagnosis, disease prognosis, and prediction of therapeutic responses. Although some miRNAs have been found to be associated with proliferation, invasion, and prognosis of PDAC, the precise mechanisms controlling the processes mentioned earlier remain elusive. Further investigation should explore the interpretation of miRNA profiling data and regulatory functions of miRNA in pancreatic cancer development and progression.

So far, the researches on miRNA's potential clinical usage are mainly conducted at molecular level and retrospectively with relatively small sample sizes. Although the results are promising, large scale prospective validation studies to test its diagnostic and prognosis value are needed. Therefore, miRNAs can serve as a valuable diagnostic marker and therapeutic target for pancreatic cancer in the future.

## Figures and Tables

**Table 1:** Important miRNAs deregulated in pancreatic ductal adenocarcinoma.

miRNA	Expression status	Target genes	Potential clinical value*	Reference
miR-21	Upregulation	PTEN, PDCD4, TPM1, TIMP3	D, P, S, T	[22–27]
miR-221/222	Upregulation	CDKN1B (p27), PUMA, PTEN	D, P, T	[22, 23, 28–30]
miR-155	Upregulation	TP53INP1, SEL1L	D, P	[22–24, 31, 32]
miR-196a	Upregulation	HOXB8, ANXA1, HMGA2	D, P	[22, 23, 33–36]
miR-424-5p	Upregulation	SOCS6	P	[28, 37]
miR-10a	Upregulation	HOXA1	P, T	[38]
miR-373	Upregulation	TP53INP1, LATS2, CD44	D	[39]
miR-27a	Upregulation	Spry2	P, T	[40]
miR-210	Upregulation	HOXA1, FGFRL1, HOXA9	P	[24, 32, 33, 41, 42]
miR-15b	Upregulation	CCNE1	P	[28, 34]
miR-181	Upregulation	TIMP3, TCL1	C	[22, 23]
miR-148a, b	Downregulation	DNMT3b, Mtif, CCKBR, BCL2	D	[22, 43, 44]
miR-198	Downregulation	MSLN, PBX-1, VCP	P, T	[45]
miR-146a	Downregulation	TRAF6, IRAK1, Stat1	T	[46]
miR-20a	Downregulation	Stat3	T	[47]
miR-96	Downregulation	KRAS	T	[48]
miR-375	Downregulation	PDK1, 14-3-3zeta	D	[22, 33]
miR-200c	Downregulation	MUC4, MUC16	P, C, T	[49, 50]
Let-7	Downregulation	KRAS, MAPK	T	[51]

*D: biomarker for diagnosis, P: predictive value for prognosis, C: indicator for chemosensitivity, T: potential target for treatment.

PTEN: phosphatase and tensin homolog, PDCD4: programed cell death 4, TPM1: tropomyosin 1, TIMP3: tissue inhibitor of metalloproteinases 3, CDKN1B (p27): cyclin-dependent kinase inhibitor 1B, PUMA: p53 upregulated modulator of apoptosis, TP53INP1: tumor protein 53-induced nuclear protein 1, SEL1L: Sel-1-like, HOXB8: Homeobox B8, ANXA1: annexin A1, HMGA2: high-mobility group AT-hook 2, SOCS6: cytokine-induced signaling 6, LATS2: large tumour suppressor homolog 2, Spry2: Sprouty2, HOXA1: Homeobox A1, FGFRL1: fibroblast growth factor receptor-like 1, HOXA9: Homeobox A9, CCNE1: cyclin E1, TCL1: T cell leukemia/lymphoma 1, DNMT3b: DNA methyltransferase 3b, Mitf: microphthalmia associated transcription factor, CCKBR: cholecystokinin-B receptor, BCL2: B cell lymphoma 2, MSLN: mesothelin, PBX-1: Pre-B-cell leukemia homeobox factor 1, VCP: valosin-containing protein, TRAF6: TNF receptor-associated factor 6, IRAK1: interleukin-1 receptor-associated kinase 1, Stat1: signal transducer and activator of transcription 1, Stat3: signal transducer and activator of transcription 3, PDK1: 3-phosphoinositide dependent protein kinase-1, MAPK: Mitogen-Activated Protein Kinase.
